# Virtual dentistry strategy to enhance knowledge, attitudes, and practices in selecting sweetened ultra-processed foods

**DOI:** 10.1371/journal.pone.0312288

**Published:** 2024-11-15

**Authors:** María del Pilar Angarita-Díaz, Johao Alexander Colmenares-Pedraza, Johanna Carolina Arias-Ramirez, Claudia Liliana Cabrera-Arango, Cristhian Ariel Cisneros-Hidalgo, Sharon Daniela Muñoz-Espitia, Jeisson Armando Garzon-Baquero

**Affiliations:** 1 Facultad de Odontología, Universidad Cooperativa de Colombia, Villavicencio, Colombia; 2 Food Security and Nutrition Dimension, Public Health Department, Villavicencio Health Secretariat, Villavicencio, Colombia; University of Petra (UOP), JORDAN

## Abstract

Education on the selection of low-sugar ultra-processed foods (UPF) plays a crucial role in promoting good oral health. This study aimed to assess the impact of a virtual educational strategy, developed in the field of dentistry, on improve Knowledge, Attitudes, and Practices (KAPs) related to oral health and the appropriate selection of sweet-tasting UPF. A quasi-experimental study with a pre-test and post-test design was conducted, involving parents and caregivers of children and pre-adolescents. Thirteen virtual learning objects (VLO) were delivered via Facebook. To measure the impact of the strategy, a validated instrument was used before the intervention, immediately after, and again six months later to assess Knowledge, Attitudes, and Practices (KAPs) on the subject. The median scores before and after the intervention were compared using the Friedman test for related samples, followed by multiple comparisons with Dunn’s test. Participants’ KAP levels were analyzed using Pearson’s Chi-square test and multiple comparisons of column proportions with Bonferroni correction. Finally, a satisfaction and applicability survey was conducted. A significant improvement (p < 0.01) was observed in the participants’ median KAP scores both immediately after the intervention and six months later. A greater number of participants reached the highest level in knowledge (Before (B): 43.8%, Immediately After (IA): 86%, After six months (A6): 81.3%), attitudes (B: 34.2%, IA: 69.4%, A6: 65.2%), and practices (B: 22.5%, IA: 53.7%, A6: 47.3%). Most participants described the course as consistently dynamic, well-organized, and appropriate timing. They also expressed their intention to apply the knowledge in their daily lives. The implemented strategy improved participants’ KAPs concerning oral health and the appropriate selection of sweet-tasting UPF. It also resulted in participants’ children consuming fewer sweet bakery products, candies, and flavored milk. Additionally, more participants reported consistently reading food labels after the intervention, although this behavior was not adopted by the majority.

## Introduction

As macro and micronutrients are essential for the development and maintenance of soft and hard tissues, nutrition and diet play a vital role in the health and integrity of the oral cavity [1, 2]. It is for this reason, that the World Health Organization (WHO), the International Dental Federation, and other organisations such as the Academy of Nutrition and Dietetics advocate for the implementation of healthy diets that support oral health [1–3].

Furthermore, the high consumption of nutrients like sucrose or other fermentable carbohydrates contributes to the occurrence of oral diseases such as dental caries, a highly prevalent non-communicable condition worldwide [4, 5]. Frequent consumption of sucrose promotes the growth of cariogenic bacteria, which metabolize it effectively, releasing acids that disrupt the balance of the oral ecosystem and lead to demineralization of the tooth enamel [6, 7].

Global data shows a high consumption of various types of sugars [8, 9], largely driven by the intake of ultra-processed foods (UPF). These foods often expose individuals to excessive levels of sugars, sodium, and saturated fats [10–12]. In Latin America, a report by the Pan American Health Organization (PAHO) recorded an 8.3% increase in UPF sales between 2009 and 2014 [13]. A more recent PAHO report highlights that individuals consuming one or more UPF products are two to four times more likely to follow inadequate dietary patterns [14]. In Colombia, individuals aged 15 to 65 consume an average of 109.8g of total sugars per day, accounting for 20.9% of total energy intake, with 59.5g per day coming specifically from added sugars [15].

Studies in Colombia reveal a high availability of UPF foods rich in critical nutrients in supermarkets [16, 17], along with high levels of childhood consumption of carbonated beverages, candies, processed chocolates, flavored drinks, and desserts [18]. For example, between 2015 and 2016, children under five consumed sweets an average of five times per week, while those aged six to twelve consumed them six times per week [18]. Furthermore, between 2005 and 2017, there was a substantial increase in the consumption of products such as desserts, confectionery, snack bars, and sweet bakery items [19].

Food literacy enhances the understanding of information about food and nutrition. However, literacy is influenced by economic, psychosocial, and cultural factors, which is why strategies have been developed to make food selection easier [20]. To promote healthier food choices, strategies such as nutritional labeling have been introduced, providing information on composition, ingredients, quantities, origin, processing, and preservation of foods [21]. Another approach is front-of-package warning labeling, a simple tool indicating whether the UPF is high in critical nutrients [22]. However, the use of these tools is not widespread due to factors including lack of awareness, limited understanding of the information, cost, brand preferences, and the limited time consumers have to review the labels [23–26]. A 2019 study by Colombia’s National Institute of Health (INS) found that only 28.1% of the surveyed population regularly checks the nutritional information on food labels, while 48.9% do so occasionally. The study also highlights widespread confusion about nutritional information, including difficulties understanding units of measurement and how nutrients contribute to overall health [23]. Front-of-package warning labels were only recently implemented in Colombia, starting in 2021 [27, 28].

Parents and caregivers of children are a crucial target group for preventive health interventions, as their eating habits significantly influence children’s behavior [29]. Studies have shown that caregivers’ knowledge, attitudes, and practices significantly influence their understanding of sugar’s effects on oral health, as well as their intention to regulate sweet snacks and beverages [30], opt for foods with lower sugar content [31], and mitigate oral health issues in children [32]. Moreover, there are strong positive correlations between nutrition knowledge and the likelihood of making healthier food choices [33].

These findings highlight the importance of implementing educational strategies to improve understanding, attitudes, and practices related to nutrition, oral health, and food choices, leveraging both nutritional labeling and front-of-package warning labels.

Given that the Internet is the primary source of health information, it is essential to create and disseminate reliable resources [34]. Information and communication technologies have provided opportunities to implement educational strategies that reach a broad audience [35]. For example, virtual education, when approached from a pedagogical perspective, enables the formation of learning communities [36] that can be established on digital platforms like social networks. These networks, powered by Web 2.0 technology, allow participants to share resources such as VLO [37, 38].

One such social media platform is Facebook, which offers features that support asynchronous education [39]. It enables the sharing of virtual resources, is easily accessible across a range of electronic devices [39], and is visited daily by nearly 2.96 billion monthly active users worldwide [40]. In Colombia, Facebook is the most popular social network, boasting approximately 31.8 million active users, predominantly in the age brackets of 25 to 34 years (29.4%), 18 to 24 years (24.3%), and 35 to 44 years (20%) [41]. This platform has proven to be an effective tool for disseminating knowledge [42], enhancing health literacy [43], and fostering community engagement [44, 45]. Moreover, Facebook has been used for dietary interventions, leading to improvements in nutritional knowledge and eating behaviors [46]. Therefore, implementing an educational strategy developed within the field of dentistry through this social network can offer valuable information on oral health, the proper selection of sweet-flavored foods, and understanding nutritional labeling. This study aims to answer the following research questions: What is the impact of an educational strategy delivered via Facebook on knowledge, attitudes, and practices related to oral health and sweet-food selection? And what is the level of participant satisfaction with the educational strategy?. The objective of this study was to assess the impact of a virtual educational strategy, implemented through Facebook, on improving the knowledge, attitudes, and practices related to oral health and the appropriate selection of sweet-tasting UPF among parents and caregivers of children and pre-adolescents.

## Materials and methods

### Study design and study population

This study received approval from the ethics subcommittee at Universidad Cooperativa de Colombia (BIO193). Participants provided written informed consent via the Google Forms platform during the recruitment period (April to May 2023). The study employed a quasi-experimental design with a pre-test and post-test, without a control group. An educational strategy was delivered via Facebook in May 2023. A total of 151 parents or caregivers of children aged 1 to 12 in Villavicencio, Colombia, participated in the study, with 121 remaining engaged throughout the strategy (a retention rate of 80.1%). The sample size calculation was based on estimating statistical power using Epidat 3.1 software (Xunta de Galicia/PAHO-WHO) for a paired sample (pre- and post-educational strategy) of the 121 participants selected through convenience sampling and who stayed engaged throughout the strategy. Assuming a 95% confidence level, an pre-test standard deviation of 0.11, a post-test standard deviation of 0.13, and a mean difference of 0.67 (1.60–0.93) for the item "When shopping, do you use the nutrition label to decide what food to buy?" from the study by Neuenschwander et al. [47], the result indicated a statistical power of 100%.

The selection criteria for participation in the study included voluntary and committed engagement in the activities, having a Facebook account, and accepting Facebook’s privacy policies. Exclusion criteria included illiteracy, cognitive disability, or possessing advanced education in medicine, dentistry, nutrition, or nursing.

### Study phases

In the initial phase, participants were recruited through social media, local educational institutions, and personal contacts (such as family, and friends). Once they accepted, they were added to a WhatsApp® group, where they received the links to the informed consent form and the questionnaire titled "Knowledge, attitudes, and practices regarding nutritional label reading and its impact on oral health among parents and caregivers of children and pre-adolescents." Finally, they were provided with the link to join the Facebook group Selección adecuada de alimentos con sabor dulce (Selecting Sweet Foods Wisely).

The course began in the second phase, where VLO were shared weekly over a four-week period (Table 1). At the end of each week, the corresponding VLO were uploaded according to the scheduled sequence. Participants were encouraged to ’like’ the videos after viewing them to help track engagement and progress. Once the strategy period concluded, an additional 2 weeks were provided for participants to view any remaining VLO.

**Table 1:** Course topics and virtual resources developed via social media.

Week	Topics	Purpose	Virtual Learning Objects
**1**	How sugar impacts human health	Explaining what sugar is and how it impacts human health.	■ Interactive infographics converted to video ■ 3 minutes
	Oral health care	Teaching about nutrition and oral health from a dental perspective.	■ Narrative/explanatory video ■ 2 minutes and 22 seconds
	The great Streptococcus attack	Explaining the effect of excessive sugar consumption on the oral microbiota.	■ Narrative/explanatory video ■ 4 minutes
**2**	Story on the impact of sugar on oral health	Raising awareness among parents about the effects of sugar on oral health.	■ Podcast or audio narrative ■ 2 minutes and 45 seconds
	The ABC of the nutritional table	Describing and explaining the nutritional information on food labels.	■ Infographics converted to video ■ 1 minute and 33 seconds
	Five keys to understanding the nutrition table	Teaching how to properly read and interpret food labels.	■ Narrative/explanatory video ■ 2 minutes and 19 seconds
**3**	Food microcapsule	Explaining the WHO recommendation on reducing sugar intake.	■ Narrative/explanatory video ■ 1 minute and 35 seconds
	Nutritional tables	Teaching proper processed food selection based on the nutritional table.	■ Video lesson ■ 2 minutes and 22 seconds
	Front warning labelling	Explaining front warning labelling.	■ Carousel of images converted to video ■ 48 seconds
**4**	Misleading advertising	Explaining misleading advertising.	■ Video newsreel ■ 2 minutes and 20 seconds
	Healthy lunchbox: practical recipes	Teaching healthy and easy-to-prepare recipes for a low-sugar lunchbox.	■ Video stop motion ■ 1 minute and 35 seconds
	Top 5: Children’s programs on food	Recommending children’s programs on healthy low-sugar eating.	■ Video ■ 3 minutes and 29 seconds
	Sugar concentration in ultra-processed sweet-tasting foods	Revealing the amount of sugar (number of spoons) in ultra-processed foods sold in supermarkets.	■ Presentation converted to video ■ 2 minutes and 27 seconds

In the third phase, participants were asked to complete the immediate impact measurement questionnaire again, along with a survey to evaluate their satisfaction and the course’s practicality. Six months later, the questionnaire was re-administered to assess the long-term impact of the strategy. Participants who encountered difficulties at any stage of the study received support via WhatsApp or phone calls.

### Educational strategy

The virtual strategy included 13 VLO, developed using insights gathered from focus groups with parents and caregivers of children aged 1 to 12 in Villavicencio [48], and aligned with Resolution 810 of 2021, which details regulations on the nutritional labeling and front-of-package warning labels for food products [27]. These VLO were created using the microlearning methodology and incorporated multimedia elements. The resources were evaluated by experts from a range of disciplines including pediatric dentistry, psychopedagogy, nutrition, epidemiology, biological sciences, social sciences, and educational and social development. These experts provided feedback and suggestions for improvement. The designs were reviewed and refined by a copyeditor [48].

Finally, the VLO were developed using various software programs (Animaker®, Powtoon®, Canva®, Genially®, InShot®, Stop motion shop®, Clipchamp®). A pilot test was conducted on Facebook with 20 parents, who rated the virtual resources and participated in interviewed to provide feedback for refining the strategy. Based on the test results, all the VLO were adapted into video format and uploaded to YouTube for easier access and viewing [49].

### Measurement instruments

This questionnaire was designed and validated by experts in the field, through a pilot test and quantitative validation [50]. The questionnaire was previously validated via a focus group, an expert panel, a pilot test, and a parent survey to determine internal consistency (K Richarson > 0.70). The questionnaire demonstrated a strong biserial index of 0.2–1 and a high discrimination index of 0.3–0.39 for most questions. Additionally, the non-response rate was within a tolerable range of 0–0.29. The difficulty index indicated that seven questions were “very easy” (0.75–1), while five were categorized as “very difficult” (0.00–0.24).

The instrument comprised 20 questions distributed across the following sections: 1. Sociodemographic data; 2. Knowledge about *sugar and oral health, *nutritional labeling, *front-of-package warning labeling, *reading nutritional labeling and its importance in oral health; 3. Attitudes rowards the importance of reading nutritional labeling and the reason for consulting labeling when doing so; 4. Practices regarding *reading nutritional labeling, *frequency of reading labeling, *correct reading of nutritional labeling, *frequency of consumption of sweet-tasting foods, and *recommendation from the dentist [50] (S1 Appendix).

Each question from the knowledge, attitudes, and practices sections was scored and classified according to the number of correct answers as follows: For the knowledge section: “low” for 0–1 correct answers, “intermediate” for 2 correct answers, and “high” for 3–6 correct answers. For the attitudes section: “negative” for one correct answer and “positive” for two correct answers. For the practices section: “poor” for 0–2 correct answers, “moderate” for 3–4 correct answers, and “good” for 5–12 correct answers.

Finally, following the implementation of the course, the questionnaire included closed-ended questions assessing satisfaction and applicability. These questions were pre-coded and utilized a five-point Likert scale, ranging from ’Always’ (5 points) to ’Never’ (1 point).

### Statistical analysis

The data analysis was conducted using IBM SPSS version 27.0. A descriptive analysis was performed by estimating relative and absolute frequencies for categorical variables and summary measures for quantitative variables (central tendency, dispersion, and position). The sociodemographic data, calculated frequencies of KAP levels, as well as course satisfaction and applicability were also analyzed. Additionally, descriptive analyses were performed using the accuracy score to determine measures of central tendency, such as the mean and median, as well as measures of dispersion, including standard deviation, and position, such as quartiles and percentiles. The strategy’s effectiveness was assessed by comparing the median of correct answers before and after the intervention (both immediately and at six months) using the Friedman test for related samples and multiple comparison analysis through Dunn’s test. Normal distribution compliance was assessed using the Kolmogorov-Smirnov test with Lilliefors correction. To compare categorized score frequencies across the three time points, Pearson’s Chi-square test was applied, followed by a multiple comparison analysis of column proportions using Bonferroni correction. A significance level of 5% was used for all statistical analyses.

## Results

### Sociodemographic variables

**Fig 1:**
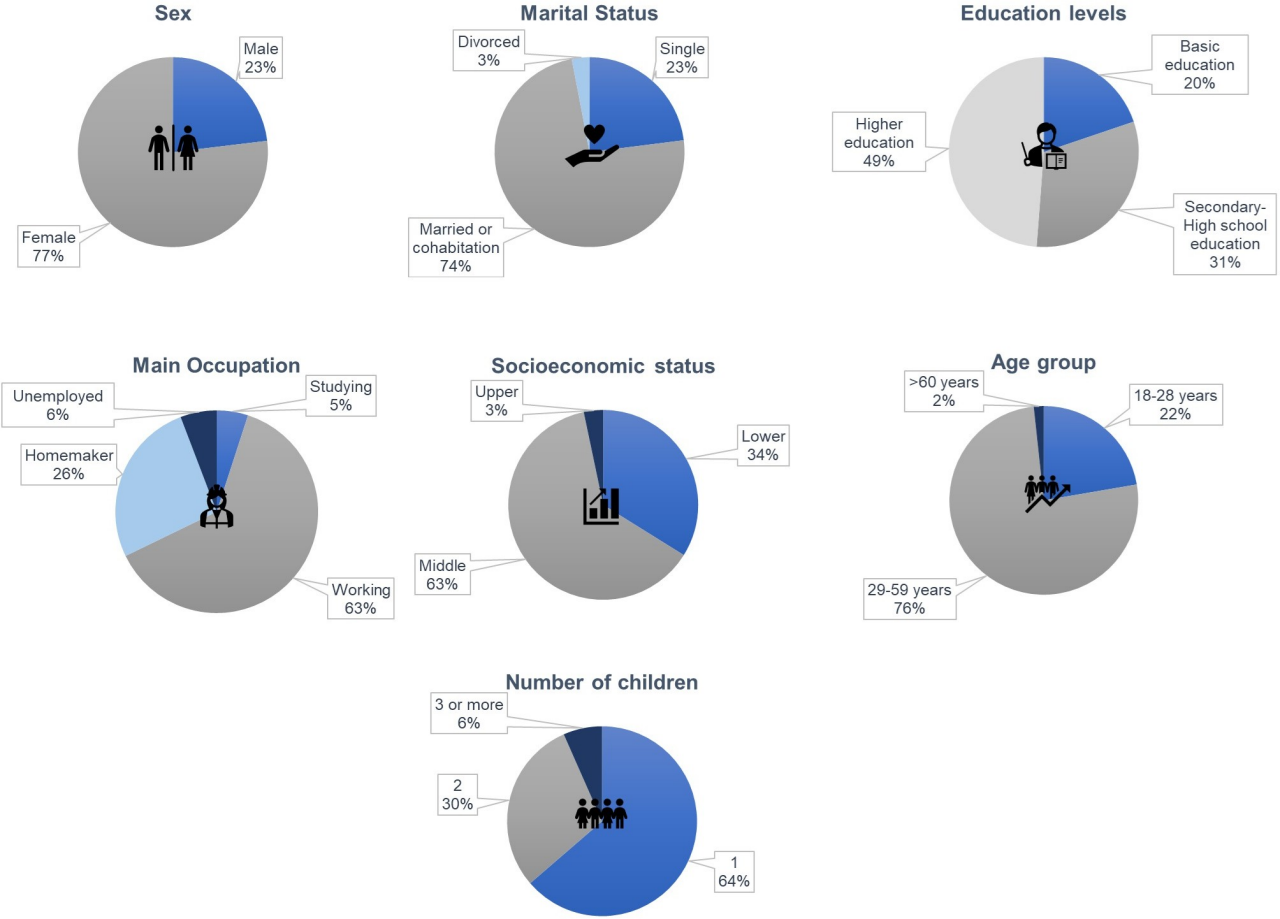
Sociodemographic variables of study participants.

The majority of participants were women (77%), married or in common-law unions (74%), had higher education (49%), were employed (63%), had a mid-level economic status (63%), were between 29 and 59 years old (76%), and had only one child between the ages of one and twelve (64%) (Fig 1).

**Fig 2:**
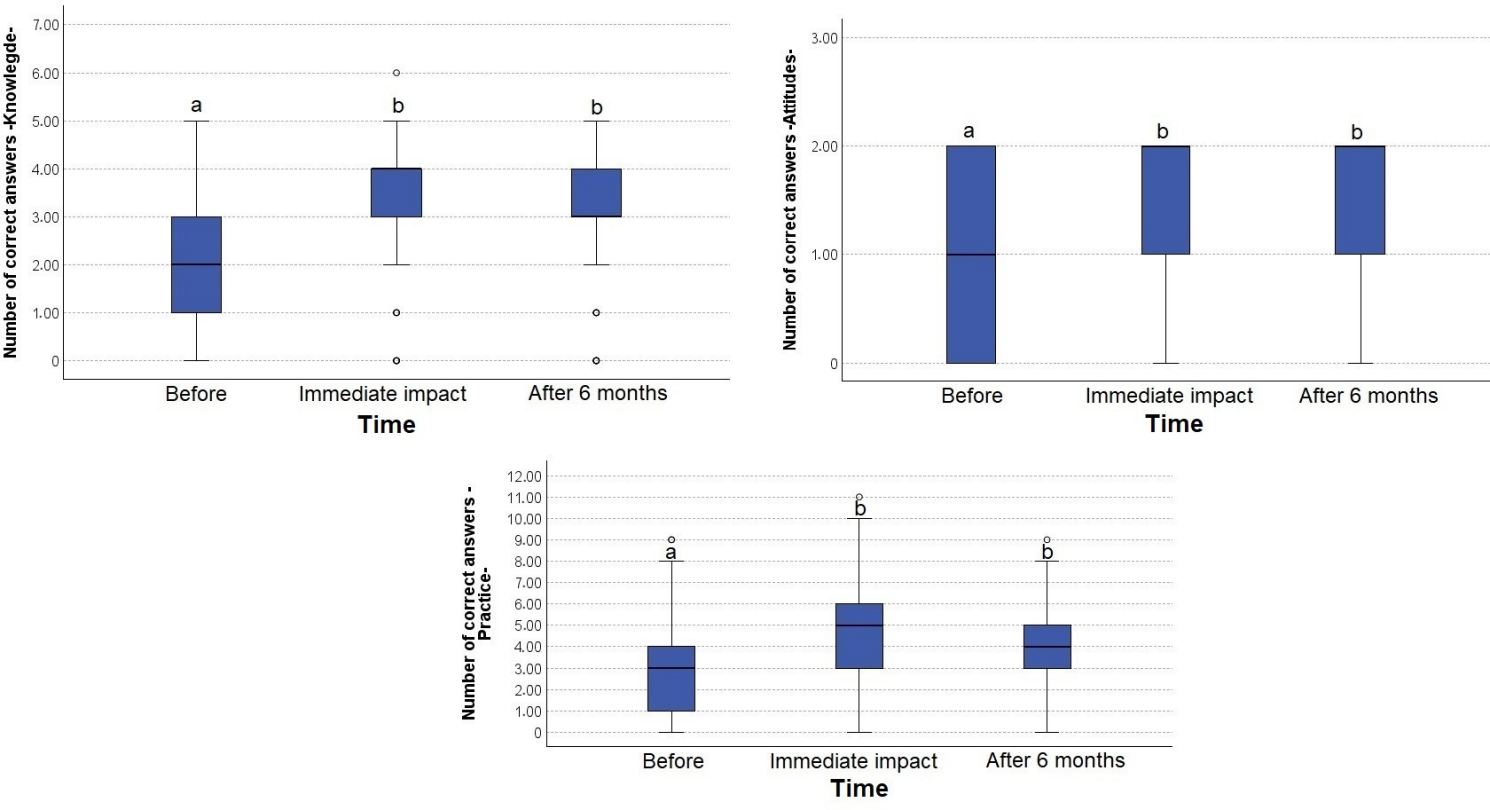
Impact of the virtual course on the number of correct answers. The numbers of correct answers on knowledge, attitudes, and practices, assessed before questionnaire application, immediately after, and 6 months post-intervention. Median values and interquartile ranges are presented. Friedman test p < 0.001. a,b: Post hoc analysis using Dunn’s test, values with different labels show statistically significant differences (p < 0.001).

**Fig 3:**
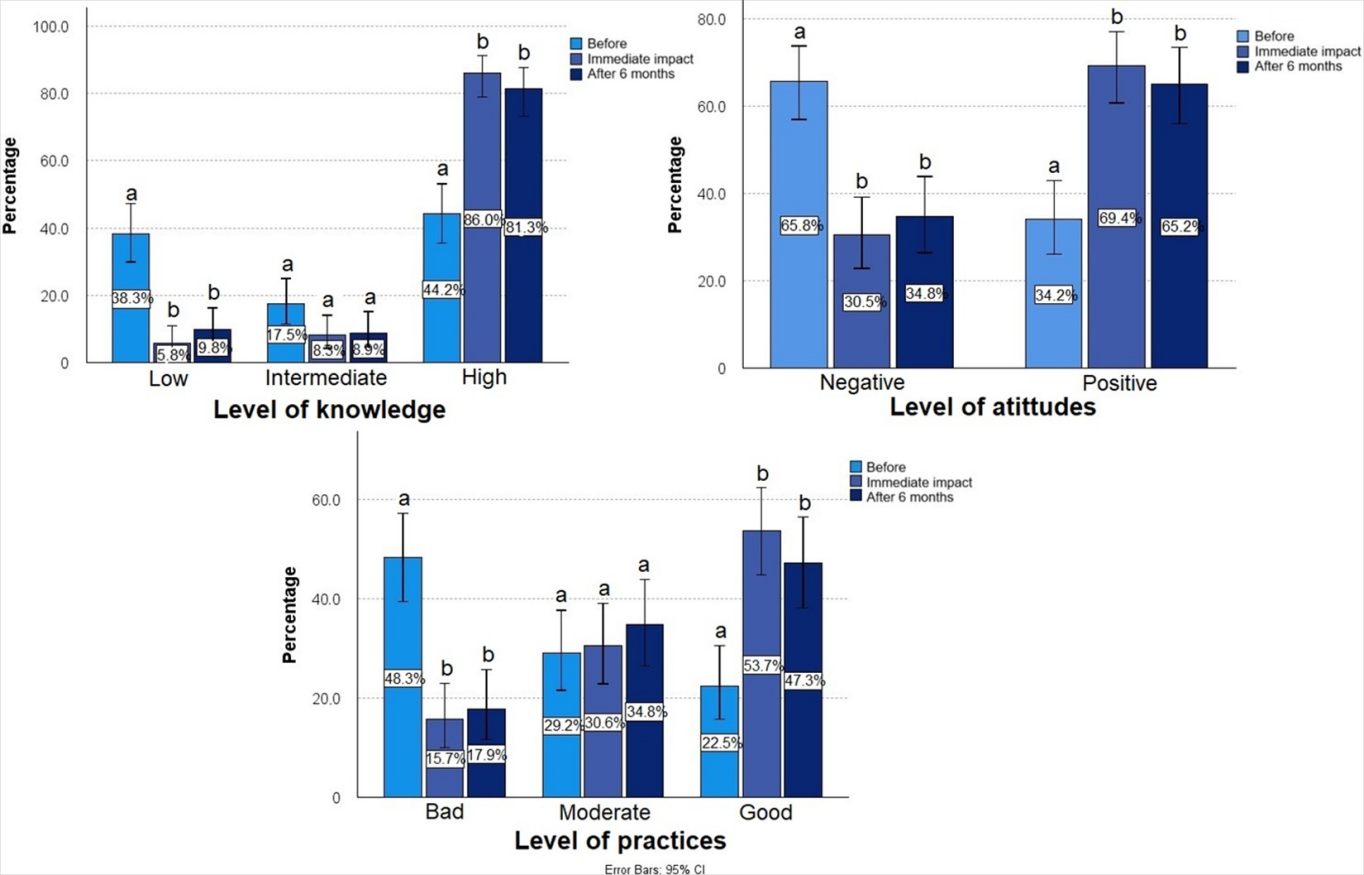
Impact of the virtual course on levels. Percentage of participants showing low, intermediate, or high levels of knowledge, negative and positive attitudes, and bad, moderate and good levels of practice, before the application of the questionnaire, immediately after, and 6 months post-intervention Pearson’s Chi-Square, a,b: multiple comparisons of column proportions, values with different labels show statistically significant differences (p < 0.001).

**Impact of the strategy on knowledge about nutritional labeling and selection of sweet foods.** Immediately following strategy implementation, the median score for correct answers increased significantly (pre-implementation: 2.0 interquartile range [IQR] [1.0–3.0], post-implementation: 4.0 IQR [3.0–4.0], p < 0.001). Even six months after the strategy’s introduction, the median score remained significantly higher compared to the beginning of the study (after: 3.0 IQR [3.0–4.0], p < 0.001) (Fig 2). The knowledge attained by the majority of participants included an understanding of nutritional labeling, familiarity with front-of-package warning labels, and awareness of the WHO-recommended sugar intake guidelines to reduce the risk of cavities (S1 Appendix). The correct identification of the sugar concentration in foods high in this ingredient also increased, although it did not impact the majority of participants. Most participants maintained a high level of knowledge, both immediately after the strategy was implemented (86.0%) and at the six-month follow-up (81.3%) (Fig 3).

#### Impact of the strategy on attitudes towards nutritional labeling and the selection of sweet foods

There was a significant increase in the median score of correct answers immediately after strategy implementation (before: 1.0 IQR [0.0–2.0], after: 2.0 IQR [1.0–2.0], p < 0.001). After six months, the score remained significantly higher than at the beginning of the study (after: 2.0 IQR [1.0–2.0], p < 0.001) (Fig 2). There was also an increase in the number of participants who considered reading the nutritional label important, citing the reason of choosing healthier products (S1 Appendix). Most participants exhibited positive attitudes, both immediately (69.4%) and at the six month mark (65.2%) (Fig 3).

#### Impact of the strategy on practices towards nutritional labeling and the selection of sweet foods

The median score increased immediately after strategy implementation (before: 3.0 IQR [1.0–4.0], after: 5.0 IQR [3.0–6.0], p < 0.001). Six months later, a significant increase in the median score was still observed (4.0 IQR [3.0–5.0], p < 0.001) (Fig 2). Both immediately and six months after strategy implementation, most participants reported receiving information about sugar quantities from their dentist, affirming that they checked nutritional labels and the sugar content in ultra-processed foods. They also successfully completed a nutritional label reading exercise. Additionally, there was an increase in the number of participants who "always" check the nutritional labels. Regarding the consumption of sweet foods, children or pre-adolescents reduced their intake of sweet bakery items such as cookies, cakes, and sweet bakery products. A decrease was also noted in the consumption of sodas, juices, flavored milk, sugared cereal, candies. The greatest reduction in consumption was observed with sweet bakery products, candies, and flavored milk (S1 Appendix). Finally, a significant increase was observed in the number of participants who achieved a good level of positive practices, both immediately after strategy implementation (53.7%) and six months later (47.3%) (Fig 3).

#### Course satisfaction and applicability

**Fig 4:**
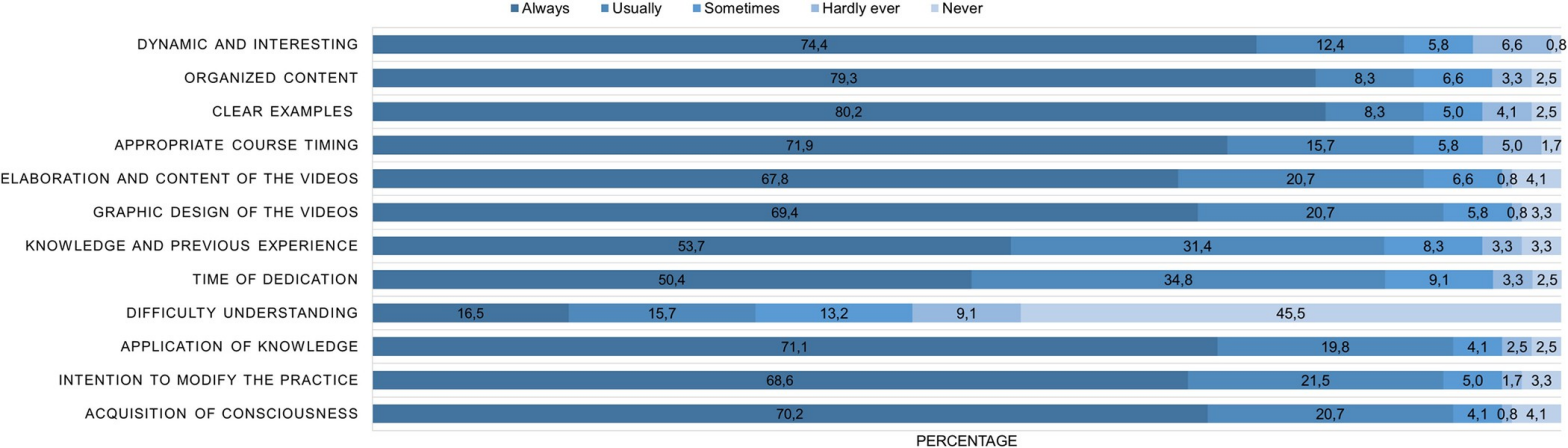
Level of satisfaction and applicability of the virtual course.

The majority of the participants described the course as "always" dynamic and interesting (74.4%), well-organized (79.3%), with clear examples (80.2%), and providing sufficient time to review the content (71.9%). Regarding the VLO, most participants "always" considered them well-constructed and conducive to learning (67.8%), with an attractive and clear graphic design (69.4%). Additionally, 45.5% indicated they "never" had difficulty understanding the resources. Finally, the majority "always" felt they could apply the knowledge gained in their daily lives (71.1%), believed the educational strategy would change the way they select foods (68.6%), and reported that the course made them more mindful when choosing sweet foods (70.2%) (Fig 4).

## Discussion

The strategy implemented for parents and caregivers of children and pre-adolescents had a significantly favorable impact on knowledge, attitudes, and practices regarding oral health and the selection of sweet-flavored UPF. These favorable results may be attributed to the type of strategy and content specifically developed for the participant population [20]. The usefulness of these web-based strategies [51, 52], particularly those delivered on social media platforms [46, 53], has proven to be valuable for educating the public on health-related topics and promoting behavioral improvements. These tools have also helped overcome certain limitations commonly encountered in face-to-face interventions, such as travel, scheduling coordination, and other logistical challenges [46].

This study also highlighted the advantages of these strategies, including low costs, ease of use of the VLO, and the wide dissemination of information to a large audience [–51–53]. Additionally, some of the barriers encountered when using Facebook—such as not having an account on the platform, a lack of understanding of privacy policies, or difficulties in accessing the learning resources—were overcome through continuous support provided to the participants [46].

Regarding oral health, most participants acknowledged from the outset that they were aware of the impact of sugar has on oral health. However, the majority reported that they had not received guidance from the dentist on the recommended daily sugar intake for their children to help prevent cavities. This gap in information may be attributed to dentists’ limited awareness or lack of expertise in incorporating preventive dietary advice into their practice [54]. Nutrition education is often undervalued during dental training [55], despite recommendations from organizations such as the US Academy of Nutrition and Dietetics [1]. Following strategy implementation, more participants reported receiving such information from their dentist, demonstrating that such strategies can effectively support oral health-related KAPs.

Despite the increase in correct responses regarding the maximum amount of sugar that can be consumed, most participants were unable to recall the numerical data related to sugar concentration that was presented on the course to raise awareness regarding UPF selection. This may be attributed to the lack of a VLO specifically designed to reinforce this numerical data, which could aid in understanding, encoding, and memorizing this information [56]. For example, strategies rooted in psychological theory can enhance cognition and the structuring of information [57]. These findings highlight the greater effectiveness of visual nutritional information compared to numerical data, such as front-of-package nutrition labels, which provide a clearer, more accessible source of information for selecting UPF [58, 59].

Before the intervention, most participants reported being familiar with and understanding nutritional labels, as well as recognizing their importance when selecting healthier products. After the intervention, nearly all participants demonstrated this knowledge and attitude. Additionally, a greater number of participants were able to correctly interpret the sugar concentration during the exercise, and more of them now "always" read nutritional labels.

However, it should be noted that the strategy did not succeed in encouraging the majority of participants to "always" read nutritional labels. This highlights that simply having knowledge and positive attitudes about nutritional labeling is not enough to ensure its consistent use. In a systematic review, Campos et al. highlighted that nutritional labeling is viewed by a significant portion of the population as a necessary and trustworthy source for making food choices [25]. However, its usage is influenced by factors that go beyond knowledge and attitudes, including lack of time to read labels, lack of interest, food costs, low income, and cultural background, among others [23–25, 60]. For example, a study by the INS in Colombia found that most surveyed individuals tend to base their choices on price and brand loyalty, often following traditional consumption patterns [23].

It is also important to highlight the significance of the current strategy in educating people about front-of-package warning labels, especially as their use was only recently mandated in Colombia [27, 28]. Countries that have employed this tool for a longer period have demonstrated its effectiveness. For instance, in Chile, where front-of-package labeling was introduced just two years ago, it has had a positive impact: 92.9% of the population now understands the information provided, and nearly 50% actively use it, leading to a reduction in purchases of high-sugar and high-sodium foods [61].

In this study, although the majority of participants were aware of sugar consumption and its effects on oral health, it was found that their children consumed UPF with high sugar content more than once a month, including items like soft drinks, boxed or bottled juices, sweet bakery goods, and candies. However, it is important to note that following the intervention, there was a decrease in the consumption of soft drinks, juices, flavored milk, candies, and sweet bakery products. These results may be attributed to the use of visually engaging resources designed for the whole family, which likely enhanced their enjoyment of the activity. Adult learning theory highlights the role of enjoyment in behavior change [62]. Other educational strategies for nutrition have also demonstrated a favorable impact on the dietary behavior of children and pre-adolescents, contributing to the reduction of critical nutrients that negatively affect health [63–65].

In terms of course satisfaction and practicality, most participants (between 70% and 80%) consistently found the training process, VLO, and the information provided to be suitable and valuable. In the review by Da Cunha et al., a favorable effect on knowledge and dietary behaviors was observed in the majority of the analyzed studies (78%). However, due to the heterogeneity of the studies, it was not possible to establish a direct link between the results and the use of Facebook as a social network. Nevertheless, Facebook did show a positive impact on participant retention and acceptability [46]. Therefore, interventions delivered through social networks like Facebook not only show promising impacts on dietary and nutritional behavior [46, 53] but also serve as valuable tools with high acceptability and utility for healthcare professionals [46].

The study had several limitations. First, the sample selection method was based on convenience sampling, and there was no control group to compare the impact of the strategy in a face-to-face setting. Moreover, it was noted that there is a need for VLOs designed to more effectively strengthen data retention, thereby increasing awareness during the selection of UPF. Participants needed reminders to view the resources provided during the study, which may not foster autonomous learning. Thus, alternative strategies should be explored to ensure sustained virtual engagement and health education. Offering participants incentives, such as gifts, as recommended by other studies, could serve as a potential solution [46]. Another limitation was the inability to track the viewing of all resources, as some participants did not follow the instruction to "like" the content. Evidence shows that some active users on social networks exhibit silent behavior, meaning they view resources without liking or commenting [66]. Additionally, the age range of participants’ children impacted the estimation of high-sugar food consumption, due to varying dietary patterns and autonomy in food choices. However, it is important to highlight that this age group was selected due to its higher susceptibility to cavities, reinforcing the need to educate caregivers of this population.

### Conclusion

The implementation of a virtual course on the Facebook platform significantly improved the knowledge, attitudes, and practices regarding oral health and the selection of sweet-flavored UPF among parents and caregivers of children and preadolescents in Villavicencio. Moreover, there was a noticeable decrease in the consumption of sweet bakery products, candies, and flavored milk. While the strategy increased the frequency of reading nutritional labels, it did not consistently lead to participants "always" reading the labels.

## Supporting information

S1 Appendix Immediate and 6-months impact of the virtual dentistry strategy implementation. Impact on the number of participants responding to the questionnaire.
(DOCX)
