# Supplementary material for: Virtual dentistry strategy to enhance knowledge, attitudes, and practices in selecting sweetened ultra-processed foods
Source: PLoS One. 2024 Nov 15;19(11):e0312288. doi: 10.1371/journal.pone.0312288 (PMC11567513; doi:10.1371/journal.pone.0312288)
Supplement: S1 Appendix — Impact on the number of participants responding to the questionnaire. (DOCX) [file pone.0312288.s001.docx]

**S1 Appendix. Immediate and 6-months impact of the virtual dentistry strategy implementation.**Impact on the number of participants responding to the questionnaire.

| Responses | BEFORE % | AFTER% | AFTER% |
| --- | --- | --- | --- |
|  | **Before** | **Immediate impact** | **6-months impact** |
| KNOWLEDGE | | | |
| Do foods high in sugar negatively impact your child’s/children’s oral health? | | | |
| Yes | 97.5 | **99.2** 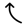 | 97.1 |
| No | 1.7 | 0.8 | 0.9 |
| Doesn’t know/Doesn’t say | 0.8 | 0 | 0 |
| According to the World Health Organization, what is the maximum daily sugar intake recommended to reduce the risk of cavities? | | | |
| 12.5 g a day (approx. three teaspoons) | 42.1 | 58.7 | 81.6 |
| 25 g a day (5 teaspoons)* | 16.5 | 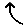**33.9** | 4.9 |
| 50 g a day (10 teaspoons) | 5.0 | 3.3 | 1.0 |
| Doesn’t know/Doesn’t say | 36.4 | 4.1 | 0 |
| Are you familiar with the nutrition facts labels on some food packages? | | | |
| Yes* | 71.9 | 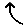**95.9** | 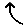**85.4** |
| No | 22.3 | 1.7 | 9.7 |
| Do you understand the information provided on nutrition facts labels? | | | |
| Doesn’t know/Doesn’t say | 5.8 | 2.5 | 4.9 |
| Yes* | 68.2 | 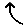**95.7** | 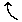**89.8** |
| No | 28.4 | 4.3 | 9.1 |
| Are you familiar with front-of-package warning labelling? | | | |
| Doesn’t know/Doesn’t say | 3.4 | 0 | 1.1 |
| Yes* | 55.4 | 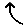**87.6** | 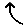**89.3** |
| No | 42.1 | 9.9 | 9.7 |
| Doesn’t know/Doesn’t say | 2.5 | 2.5 | 1.0 |
| How much sugar does a solid food, like biscuits, have to contain to be classified as high in sugar? | | | |
| 5 g in 100 g or more (one teaspoon) | 13.4 | 23.6 | 43.5 |
| 10 g in 100 g or more (two teaspoons)* | 26.9 | 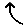**39.6** | 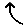**30.4** |
| 20 g in 100 g or more (four teaspoons) | 23.9 | 34.0 | 20.7 |
| Doesn’t know/Doesn’t say | 35.8 | 2.8 | 5.4 |
| How much sugar does a drink, such as a soft drink, have to contain to be classified as high in sugar? | | | |
| 5 g in 100 ml or more (one teaspoon)* | 13.4 | 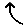**34.0** | 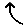**43.5** |
| 10 g in 100 ml or more (two teaspoons) | 23.9 | 26.4 | 26.1 |
| 20 g in 100 ml or more (four teaspoons) | 32.8 | 34.9 | 23.9 |
| Doesn’t know/Doesn’t say | 29.9 | 4.7 | 6.5 |
| ATTITUDES | | | |
| Do you think it is important to read nutrition facts labels before purchasing food? | | | |
| Yes* | 93,1 | 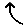**100** | 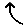**100** |
| No | 5,7 | 0 | 0 |
| Doesn’t know/Doesn’t say | 1,1 | 0 | 0 |
| What is your primary reason for reading the nutrition facts label? | | | |
| I have a disease | 0 | 1.1 | 0 |
| To choose healthier products* | 81.1 | 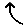**89.4** | 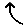**91.5** |
| Following a diet | 5.7 | 3.2 | 2.8 |
| To lose weight | 0 | 1.1 | 0 |
| Out of curiosity | 5.7 | 5.3 | 4.2 |
| Other | 7.5 | 0 | 1.4 |
| PRACTICES | | | |
| Do you read the nutrition facts labels on food packages? | | | |
| Yes* | 41.3 | 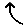**77.7** | 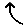**68.9** |
| No | 55.4 | 20.7 | 30.1 |
| Doesn’t know/Doesn’t say | 3.3 | 1.7 | 1.0 |
| How often do you read the nutrition facts labels on the products you find in the supermarket? | | | |
| Always* | 16.0 | 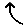**37.2** | 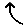**28.2** |
| Sometimes | 80.0 | 60.6 | 67.6 |
| Rarely | 4.0 | 2.1 | 4.2 |
| Never | 0 | 0 | 0 |
| Doesn’t know/Doesn’t say | 0 | 0 | 0 |
| Do you check the sugar content when reading the nutrition facts label?? | | | |
| Yes* | 84.3 | 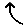**96.8** | 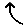**94.4** |
| No | 11.8 | 2.1 | 5.6 |
| Doesn’t know/Doesn’t say | 3.9 | 1.1 | 0 |
| Has your dentist given you recommendations on the maximum amount of sugar your child should consume to reduce the risk of cavities? | | | |
| Yes* | 38.8 | 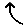**68.6** | 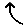**72.8** |
| No | 58.7 | 29.8 | 24.3 |
| Doesn’t know/Doesn’t say | 2.5 | 1.7 | 2.9 |
| The following figure displays the nutritional information of a dairy product. Based on the nutrition facts label, how many grams of sugar are consumed per 100 grams of the product | | | |
| 5.6 g (approx. five teaspoons of sugar) | 10.7 | 14.0 | 0 |
| 17.4 g (approx. five teaspoons of sugar)* | 51.2 | 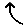**62.0** | 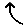**65.0** |
| 26 g (approx. five teaspoons of sugar) | 11.6 | 14.0 | 10.7 |
| Doesn’t know/Doesn’t say | 26.4 | 9.9 | 24.3 |
| Please answer the following question based on the eating habits of the 1-12-year-old child in your care. If you have more than one child in this age range, answer based on the youngest child’s habits.  How many times has the child consumed the following foods in the past month? | | | |
| Soft drinks, tea and soda (powder, carton or bottle, except for diet drinks) | | | |
| Never* | 8.3 | 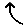**14.0** | 6.8 |
| Rarely | 33.1 | 30.6 | 37.9 |
| Once a month/ | 6.6 | 17.7 | 11.7 |
| More than once a month | 31.4 | 28.9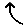 | 24.3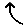 |
| One to three times a week | 13.2 | 6.6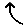 | 16.5 |
| Four to six times a week | 1.7 | 1.7 | 1.0 |
| Once a day | 2.5 | 0.8 | 1.9 |
| More than once a day | 3.3 | 1.7 | 0 |
| Doesn’t know/Doesn’t say | 0 | 0.5 | 0 |
| Juice packaged in a carton, bottle, or bag | | | |
| Never* | 13.2 | 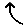**19.0** | 6.8 |
| Rarely | 36.4 | 34.7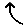 | 42.7 |
| Once a month/ | 13.2 | 17.4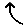 | 20.4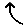 |
| More than once a month | 17.4 | 19.8 | 15.5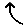 |
| One to three times a week | 9.1 | 5.8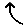 | 10.7 |
| Four to six times a week | 5.8 | 0.8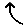 | 1.0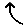 |
| Once a day | 2.5 | 2.5 | 1.9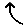 |
| More than once a day | 2.5 | 0 | 0 |
| Doesn’t know/Doesn’t say | 0 | 0 | 1.0 |
| Sweet bakery products—packaged sweet biscuits, cakes, or sweet bread | | | |
| Never | 3.3 | 3.3 | 1.0 |
| Rarely* | 19.8 | 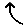**28.9** | 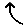**34.0** |
| Once a month* | 5.0 | 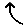**18.2** | 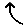**19.4** |
| More than once a month | 33.1 | 32.2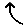 | 22.3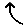 |
| One to three times a week | 18.2 | 9.9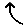 | 14.6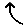 |
| Four to six times a week | 5.8 | 3.3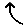 | 2.9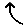 |
| Once a day | 7.4 | 3.3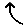 | 3.9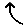 |
| More than once a day | 7.4 | 0.8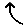 | 1.9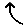 |
| Doesn’t know/Doesn’t say | 0 | 0 | 0 |
| Sugar-coated breakfast cereals (e.g., frosted cornflakes) | | | |
| Never* | 25.6 | 24.0 | 15.5 |
| Rarely | 28.9 | 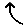**41.3** | 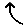**40.8** |
| Once a month/ | 11.6 | 14.9 | 13.6 |
| More than once a month | 24.0 | 17.4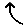 | 19.4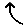 |
| One to three times a week | 5.8 | 0.8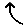 | 6.8 |
| Four to six times a week | 2.5 | 0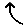 | 1.0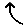 |
| Once a day | 0.8 | 1.7 | 2.9 |
| More than once a day | 0.8 | 0 | 0 |
| Doesn’t know/Doesn’t say | 0 | 0 | 0 |
| Other types of sweet yogurt (such as Greek yogurt and Kefir) | | | |
| Never | 38.0 | 35.5 | 30.1 |
| Rarely | 24.8 | 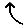**30.6** | 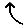**36.9** |
| Once a month/ | 13.2 | 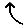**16.5** | 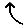**12.6** |
| More than once a month | 17.4 | 16.5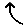 | 16.5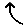 |
| One to three times a week* | 5.0 | 0 | 1.9 |
| Four to six times a week | 0 | 0 | 1.0 |
| Once a day | 0.8 | 0.8 | 0 |
| More than once a day | 0.8 | 0 | 1.0 |
| Doesn’t know/Doesn’t say | 0 | 0 | 0 |
| Sugared and flavoured milk | | | |
| Never* | 37.2 | 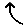**42.1** | 29.1 |
| Rarely | 20.7 | 24.8 | 36.9 |
| Once a month/ | 9.9 | 14.9 | 10.7 |
| More than once a month | 20.7 | 14.9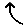 | 17.5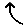 |
| One to three times a week | 6.6 | 0.8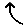 | 5.8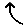 |
| Four to six times a week | 3.3 | 0.8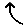 | 0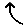 |
| Once a day | 0.8 | 1.7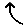 | 0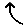 |
| More than once a day | 0.8 | 0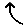 | 0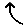 |
| Doesn’t know/Doesn’t say | 0 | 0 | 0 |
| Sweets or candy (sweets, lollipops, chocolate, jelly gums, and mints) | | | |
| Never* | 5.0 | 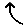**7.4** | 2.9 |
| Rarely | 24.0 | **30.6** 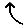 | 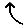**31.1** |
| Once a month/ | 7.4 | 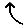**47.1** | 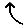15.5 |
| More than once a month | 31.4 | 0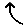 | 30.1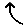 |
| One to three times a week | 19.8 | 9.9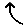 | 10.7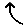 |
| Four to six times a week | 6.6 | 2.5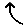 | 5.8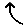 |
| Once a day | 2.5 | 2.5 | 2.9 |
| More than once a day | 3.3 | 0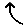 | 1.0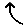 |
| Doesn’t know/Doesn’t say | 0 | 0 | 0 |

*Question scored as correct. Number in bold: Favourable increase
